# Efficacy and safety of early mobilization in patients with ARDS: a systematic review and meta-analysis

**DOI:** 10.3389/fmed.2026.1928774

**Published:** 2026-09-21

**Authors:** Zeya Wu, Xiaoyue Wei, Yan Sun, Pei Yang, Rong She, Xiu Jin, Huaping Wei

**Affiliations:** 1 School of Nursing, Lanzhou University, Lanzhou, Gansu, China; 2 School of Nursing, Gansu University of Chinese Medicine, Lanzhou, Gansu, China; 3 Department of Cardiology, The First Hospital of Lanzhou University, Lanzhou, Gansu, China; 4 Outpatient Department, The First Hospital of Lanzhou University, Lanzhou, Gansu, China

**Keywords:** acute respiratory distress syndrome, delirium, early mobilization, evidence-based nursing, hospital stay length, intensive care unit-acquired weakness, mechanical ventilation duration, Meta-analysis

## Abstract

**Objective:**

To evaluate the effect of early mobilization in patients with acute respiratory distress syndrome (ARDS).

**Methods:**

Computerized searches were conducted in PubMed, EMbase, The Cochrane Library, Web of Science, CNKI, Wanfang Data, CBM, and VIP databases for randomized controlled trials (RCTs) on early mobilization in ARDS patients. The retrieval period was from the establishment of each database to September 30, 2025. Two reviewers independently assessed the quality of included literature and extracted data in accordance with Cochrane systematic review methodology. Meta-analysis was performed using RevMan 5.4.1 software for eligible studies.

**Results:**

A total of 7 RCTs were included, consisting of 4 English and 3 Chinese articles. Meta-analysis showed that compared with conventional treatment, early mobilization significantly reduced the incidence of intensive care unit-acquired weakness (ICU-AW) [OR = 0.35, 95% CI (0.19,0.64), *P* = 0.0006] and delirium [OR = 0.51, 95% CI (0.28,0.91), *P* = 0.02], shortened the duration of mechanical ventilation [MD = −2.40,95% CI (-2.96,-1.84), *P* < 0.00001], length of ICU stay [MD = −3.36, 95% CI (−4.21,-2.52), *P* < 0.00001] and total hospital stay [MD = −2.74,95% CI (-3.49,-1.98), *P* < 0.00001]. Meanwhile, it improved patients’functional independence score (Barthel Index) [MD = 17.27, 95% CI (11.18, 23.35), *P* < 0.00001] and decreased the incidence of complications [OR = 0.39, 95% CI (0.17, 0.86), *P* = 0.02]. All pooled effects were statistically significant.

**Conclusion:**

Early mobilization yields significant clinical benefits for ARDS patients, improving short-term clinical outcomes and functional recovery. It is worthy of clinical promotion and application. However, multicenter, large-sample, and methodologically standardized RCTs are still needed to further verify its long-term effects and optimal implementation protocols.

## 1 Introduction

Research indicates that the S2e guidelines published by the German Society of Anaesthesiology and Intensive Care Medicine define early mobilization in mechanically ventilated patients (EM-MV) as rehabilitation measures involving passive and/or assisted passive or active movement initiated within 72 hours of a critically ill patient being placed on mechanical ventilation (1). The efficacy and safety of EM-EV have been confirmed by numerous studies (2–4).The current main treatment for severe ARDS is high-flow mechanical ventilation. Although mechanical ventilation plays a crucial role in saving patients’ lives, prolonged ventilation can lead to ventilator-associated pneumonia and ICU-acquired myasthenia, posing a significant threat to patients’ health and long-term quality of life (5).Research has shown (6) that early mobilization exercises can effectively preserve respiratory muscle function, reduce the risk of disuse atrophy and lower the incidence of muscle weakness. Furthermore, research has confirmed that implementing early bedside activity interventions can significantly improve the skeletal muscle metabolic status of critically ill patients and reduce the duration of mechanical ventilation (7). Ouyang Fang et al. (8) conducted early, phased pulmonary rehabilitation exercises in patients with severe ARDS, which effectively improved the patients’ pulmonary ventilation and arterial blood gas parameters, reduced the incidence of ventilator-associated pneumonia, and enhanced the patients’ quality of life. Morris et al. (9) found that physical decline is most pronounced among ICU patients with ARDS; reducing the number of days spent in bed can effectively prevent frailty, as inactivity may lead to a daily decline in muscle strength of 1.3% to 3% in healthy individuals (10). Therefore, a suitable early mobilization programme should be devised to enable ARDS patients to commence early mobilization as soon as possible. As there is currently no evidence-based data on this type of activity, it is not possible to determine from the existing literature whether early mobilization is beneficial for patients with ARDS. This study reviews relevant domestic and international literature and uses a meta-analysis to evaluate the effectiveness of early mobilization in patients with ARDS, thereby providing evidence-based guidance for clinical healthcare professionals to optimise patient outcomes.

## 2 Materials and methods

### 2.1 Literature search strategy

The following Chinese and English databases were searched: PubMed, Embase, the Cochrane Library, Web of Science, CNKI, Wanfang Data, the CBM and VIP. The search period covered the period from the establishment of each database to September 30, 2025, and all search queries combined subject headings with free-text terms. In addition to the electronic database searches, we performed supplementary citation searching to minimize the risk of missing relevant studies. Specifically, we conducted backward citation searching by manually screening the reference lists of all included studies and relevant review articles, and forward citation searching by using Google Scholar to track subsequent citations of the included studies. These complementary approaches were undertaken to identify additional potentially eligible records that were not captured by the primary electronic searches.PubMed was used as an example, and the detailed search strategy is shown in Supplementary Table 1.

### 2.2 Inclusion and exclusion criteria for literature

The inclusion and exclusion criteria of this study were established based on the PICOS framework, covering population,intervention, comparison, outcomes, and study design (11).

#### 2.2.1 Inclusion criteria for literature

(1) **Population (P)**: Patients with ARDS aged 18 years or over.
(2) **Intervention (I)**: In addition to standard treatment, early mobilization—including various passive and active exercises—should be implemented.
(3) **Comparison (C)**: The control group received standard treatment, such as mechanical ventilation and drug therapy.
(4) **Outcome (O)**: Incidence of ICU-AW, duration of mechanical ventilation, length of stay in the ICU, incidence of delirium, total length of hospital stay, functional independence score (Barthel Index), and incidence of complications.
(5) **Study design (S)**: Only randomized controlled trials were included, as this design allows for stricter control of confounding factors and improves the rigor and reliability of the findings (12).

#### 2.2.2 Literature exclusion criteria

(1) Studies without a control group were excluded.
(2) Reviews, case reports, clinical reports, conference abstracts,and other non-randomized studies were excluded.
(3) Studies with unclear outcome measures or incomplete data were excluded.
(4) Exclude grey literature, duplicate publications, and literature for which the full text is unavailable; exclude literature not in Chinese or English.

### 2.3 Literature screening and data extraction

Data extraction involved importing the retrieved literature into EndNote 21 software for duplication checking and screening. Two researchers independently reviewed the literature and, in accordance with the inclusion and exclusion criteria, independently screened the literature and extracted data. In the event of any discrepancies, these were resolved through consultation between the two researchers or by seeking the opinion of all three researchers. Data extracted included the authors, year of publication, study location, study design, sample size, control and intervention measures, measurement tools, and outcome measures, amongst others.

### 2.4 Evaluation of the quality of literature

Two researchers independently assessed the studies; where there was a disagreement that could not be resolved through discussion, a third researcher was asked to make the final decision. The researchers assessed the included studies using the risk of bias criteria set out in the Cochrane Handbook of Review Methodology (Version 5.1.0). Two reviewers independently screened all identified records based on the predefined eligibility criteria. Inter-rater agreement was assessed using Cohen‘s kappa coefficient (*κ*) at both the title or abstract and full-text screening stages. Disagreements were resolved through consensus discussion or by consultation with a third reviewer.

### 2.5 Confidence in evidence

We assessed the confidence in the evidence using the Grading of Recommendations Assessment, Development, and Evaluation (GRADE) approach for the priority outcomes (13).

### 2.6 Statistical methods

Meta-analysis was performed using RevMan 5.4.1 software. When quantitative data were collected using the same scale, the mean difference (MD) was used to pool the effect sizes; when quantitative data were collected using different scales, the standardised mean difference (SMD) was used to pool the effect sizes. If the quantitative data in the included studies were provided only as the first, third and fourth quartiles, these were converted into mean and standard deviation values before the data were pooled for analysis (14). First, a test for heterogeneity was conducted on the included studies, and the appropriate data pooling method was selected based on the results. If *P* ≥ 0.05 and I^2^ ≤ 50%, a fixed-effects model was used for analysis; if *P* < 0.05 and I^2^ > 50%, indicating substantial heterogeneity between studies, a random-effects model was used to pool the effect sizes. If heterogeneity could not be resolved, sensitivity analyses and subgroup analyses were performed. To explore the sources of heterogeneity in outcomes with substantial heterogeneity (I^2^ > 50%), we performed leave-one-out sensitivity analyses, in which each study was sequentially excluded to assess its influence on the pooled effect estimate and overall heterogeneity. For outcomes where heterogeneity was successfully resolved after excluding the identified study, the pooled results after exclusion were reported as the primary findings. For dichotomous variables, the odds ratio (OR) and 95% CI were used as measures of effect; for continuous variables, the standardised mean difference (SMD) and 95% CI were selected. Data that could not be quantitatively pooled were analysed qualitatively only. Assessment of publication bias was planned following Cochrane Handbook standards (15). Standard quantitative tools for detecting publication asymmetry include funnel plot visual judgment, Egger's linear regression test and Begg's rank correlation test (16); the trim-and-fill method can be used to adjust pooled effect sizes if significant publication bias is detected (17).

## 3 Results

### 3.1 Results of literature screening

The initial search yielded 1,082 articles, of which 4 were in English and 3 in Chinese. Following screening in accordance with the inclusion and exclusion criteria, 7 articles were ultimately included (18–24). The full study selection process is shown in the PRISMA flow diagram (Supplementary Figure 1).

### 3.2 Basic characteristics of the included

Seven RCTs (18–24) were included, involving a total of 572 patients; of these, three studies (21, 23, 24) were from North America, three (19, 20, 22) from Asia, and one (18) from Oceania. The studies were published between 2009 and 2024; four were in English (18, 21, 23, 24) and three in Chinese (19, 20, 22), with all participants recruited from hospital ICUs. There were no statistically significant differences in patient gender or age between the groups; baseline characteristics were consistent across groups. All studies described the treatment and nursing methods for both the intervention and control groups and specified clear outcome measures. The study designs were all RCTs. The basic characteristics of the included studies are shown in Supplementary Table 2.

### 3.3 Evaluation of literature quality

Of the included studies, two (21, 22) used a random number table for allocation, two (18, 24) used computerised randomisation, and three (19, 20, 23) merely mentioned random allocation without specifying the method used; Two studies (21, 22) mentioned the use of concealed envelopes, whilst the remainder did not; none of the included studies reported incomplete data or data missing. Inter-rater agreement for the quality assessment with a kappa value of Kappa = 0.85, indicating almost perfect agreement between the two reviewers. The seven included studies were of relatively high quality; details of the quality assessment are presented in Supplementary Table 4, and the risk of bias assessment for the included studies is shown in Supplementary Figures 2,3.

### 3.4 Confidence in evidence

The GRADE assessment of the certainty of evidence is presented in Supplementary Table 3.

### 3.5 Meta-analysis results

#### 3.5.1 Duration of mechanical ventilation

Seven studies (18–24) reported on the effects of early mobilization on the duration of mechanical ventilation in patients with ARDS. The heterogeneity test revealed (I^2^ = 70%, *P* = 0.003); sensitivity analysis indicated that the study by Wu Hui et al. (19) was the primary source of heterogeneity. After excluding this study, the heterogeneity test results were (I^2^ = 19%, *P* = 0.29). A fixed-effects model was therefore selected for the meta-analysis. The results showed: MD = −2.40, 95% CI [−2.96, −1.84], *P* < 0.00001. The findings indicate that early mobilization shortens the duration of mechanical ventilation in patients. See Supplementary Figure 4.

##### 3.5.1.1 Subgroup analysis by mobilization type

To further investigate the sources of heterogeneity in the duration of mechanical ventilation, we performed subgroup analyses stratified by intervention type. As shown in Supplementary Figure 5, the effect of early mobilization on mechanical ventilation duration was statistically significant in both subgroups. In the active mobilization subgroup (4 studies), the pooled mean difference was MD = −2.21 (95% CI: −3.21 to −1.21; I^2^ = 77%). In the passive mobilization subgroup (3 studies), the pooled mean difference was MD = −3.30 (95% CI: −4.26 to −2.34; I^2^ = 68%).

#### 3.5.2 Length of stay in the ICU

Five studies (18, 20–23) reported on the effect of early mobilization on the length of ICU stay in patients with ARDS. The results of the heterogeneity test (I^2^ = 34%, *P* = 0.19) led to the selection of a fixed-effects model for the meta-analysis. The results showed: MD = −3.36, 95% CI −4.21, −2.52], *P* < 0.00001. The findings indicate that early mobilization shortens the length of stay in the ICU. See Supplementary Figure 6.

#### 3.5.3 Incidence of delirium

Three studies (20, 21, 24) reported on the effect of early mobilization on the incidence of delirium in patients with ARDS; as there was a high degree of heterogeneity (I^2^ = 0%, *P* = 0.98), a fixed-effects model was used for the meta-analysis. The results showed: OR = 0.51, 95% CI [0.28, 0.91], *P* = 0.02. The findings indicate that early mobilization significantly reduces the incidence of delirium in patients with ARDS. See Supplementary Figure 7.

#### 3.5.4 Incidence of ICU-AW

Three studies (21, 22, 24) reported on the effect of early mobilization on the incidence of ICU-AW in patients with ARDS. The results of the heterogeneity test (I^2^ = 0%, *P* = 0.80) led to the selection of a fixed-effects model for the meta-analysis. The results showed: OR = 0.35, 95% CI [0.19, 0.64], *P* = 0.0006. The study indicates that early mobilization significantly reduces the incidence of ICU-AW in patients with ARDS. See Supplementary Figure 8.

#### 3.5.5 Functional independence score (Barthel Index)

Three studies (21, 22, 24) reported on the impact of early mobilization on functional independence scores (Barthel Index) in patients with ARDS. The results of the heterogeneity test (I^2^ = 85%, *P* = 0.001). Sensitivity analysis revealed that the study by Fan Dingrong et al. (22) was the primary source of heterogeneity; upon excluding this study, the heterogeneity test result was I^2^ = 0%, *P* = 0.42. A fixed-effects model was therefore selected for the meta-analysis. The results showed: MD = 17.27, 95% CI [11.18, 23.35], *P* < 0.00001. The findings indicate that early mobilization improves patients’ functional independence scores (Barthel Index). See Supplementary Figure 9.

#### 3.5.6 Total length of stay

Six studies (18, 19, 21–24) reported on the effect of early mobilization on the total length of hospital stay in patients with ARDS. The results of the heterogeneity test (I^2^ = 25%, *P* = 0.24) led to the selection of a fixed-effects model for the meta-analysis. The results showed: MD = −2.74, 95% CI [−3.49, −1.98], *P* < 0.00001. The findings indicate that early mobilization shortens the total length of hospital stay for patients. See Supplementary Figure 10.

#### 3.5.7 Incidence of complications

Three studies (19, 20, 24) reported on the impact of early mobilization on the incidence of complications in patients with ARDS. The results of the heterogeneity test (I^2^ = 66%, *P* = 0.05). Sensitivity analysis revealed that the study by Liang Ze et al. (20) was the primary source of heterogeneity; upon excluding this study, the heterogeneity test result was I^2^ = 0%, *P* = 0.55. A fixed-effects model was therefore selected for the meta-analysis. The results showed: OR = 0.39, 95% CI [0.17, 0.86], *P* = 0.02. Early mobilization significantly reduced the incidence of complications in patients with ARDS. See Supplementary Figure 11.

## 4 Discussion

### 4.1 Analysis of the methodological quality of the included studies

All seven studies included in this review were RCTs. Of these, four (18, 21, 22, 24) used a computer or a random number table for randomisation, and two (21, 22) employed allocation concealment. Outcome data were fully recorded for all studies, and there were no instances of selective reporting. Consequently, the overall quality of the included studies was moderate to high, and the results of the meta-analysis are considered to be highly reliable. However, some studies did not describe in detail the allocation concealment protocols or the implementation of blinding, which may pose a certain risk of bias. Furthermore, due to the limited number of included studies, a funnel plot analysis was not performed to assess publication bias. Consequently, there remains a need for future randomised controlled trials with more rigorous designs, involving multiple centres, large sample sizes and methodological rigour, to provide higher-level evidence-based support for the use of early mobilization in patients with ARDS.

### 4.2 An analysis of the effects of early mobilization in patients with ARDS

#### 4.2.1 The effect of early mobilization on the duration of mechanical ventilation

Research findings indicate that early mobilization in patients with ARDS shortens the duration of mechanical ventilation. A study (25) showed that the incidence of muscle weakness in patients on mechanical ventilation ranges from 35% to 50%; prolonged bed rest and mechanical ventilation not only cause systemic physiological dysfunction but also induce severe pathological changes, thereby increasing mortality rates. Implementing an early mobilization strategy can effectively reduce the accumulation of pulmonary secretions, increase tidal volume, improve lung compliance, and effectively prevent complications such as alveolar atelectasis, whilst protecting the respiratory muscles and diaphragm, thereby helping patients to be weaned off the ventilator at an earlier stage (7). A study by Wu Liangfeng et al. (26) demonstrated that the intervention group, which implemented an early ambulation programme, showed a significantly higher rate of successful weaning, as well as significantly lower rates of reintubation within 48 hours, readmission to the ICU, duration of mechanical ventilation and length of hospital stay; these findings are consistent with the results of the present study.

The subgroup analysis revealed that heterogeneity was not fully explained by intervention type. Although the I^2^ values within both subgroups remained substantial (77% and 68%, respectively), the test for subgroup differences was not statistically significant (*P* = 0.12, I^2^ = 59.2%), suggesting that the effect of early mobilization on mechanical ventilation duration did not significantly differ between active physical or occupational mobilization and pulmonary rehabilitation or passive mobilization. The persistent within-subgroup heterogeneity indicates that other clinical covariates,such as differences in ARDS severity, ventilation strategies, sedation protocols, or the timing, frequency, and intensity of the interventions, may have contributed to the observed variability. However, due to the limited number of studies, we were unable to perform additional subgroup analyses or meta-regression to further explore these potential effect modifiers.

Heterogeneity investigation was limited by the small number of included studies. Leave-one-out sensitivity analyses and subgroup analyses by intervention type did not fully resolve the heterogeneity, with an overall I^2^ of 78% for mechanical ventilation duration and within-subgroup I^2^ values of 77% and 68%. This suggests that unmeasured clinical factors including ARDS severity, ventilation strategies, sedation protocols, and intervention parameters likely contributed to the variability. The limited study numbers precluded meta-regression. Therefore, our subgroup findings are exploratory and require confirmation in future large-scale studies.

#### 4.2.2 The impact of early mobilization on length of stay in the ICU and total length of hospital stay

Research has shown that early mobilization in patients with ARDS can shorten both the duration of ICU stay and the total length of hospitalisation. The reduction in hospitalisation duration reflects the combined benefits of early mobilization. Due to prolonged bed rest and mechanical ventilation, patients with ARDS are prone to various complications, including ICU-acquired frailty, ventilator-associated pneumonia and delirium (4, 5). These complications interact with one another, creating a vicious cycle that leads to prolonged hospitalisation. Through multiple mechanisms—including the promotion of muscle function recovery, improvement in respiratory function, and a reduction in the incidence of complications (27–29)—early mobilization effectively breaks this vicious cycle and accelerates the patient's recovery process. A study by Zhou Qian et al. (30) confirmed that early mobilization can improve respiratory coordination and shorten the duration of mechanical ventilation and ICU treatment. Morris et al. (9) implemented early mobilization in patients with acute respiratory failure; the results showed that the duration of ICU stay was significantly shorter in the intervention group. Du Anqi et al. (31) pointed out that early mobilization can improve patients’ muscle strength and shorten both ICU and total hospital stays, findings which are consistent with the results of this study.

#### 4.2.3 The impact of early mobilization on the incidence of delirium

Research has found that early mobilization in patients with ARDS can reduce the incidence of delirium, consistent with the findings of Needham et al. (32, 33). Delirium is a common acute brain dysfunction in ICU patients, with an incidence of 60%–80% (7), and is closely associated with prolonged mechanical ventilation, increased mortality and long-term cognitive impairment. Due to mechanical ventilation and prolonged bed rest, coupled with physical restraints, an unfamiliar environment and a lack of family support, ARDS patients are prone to the accumulation of negative emotions, which further increases the risk of delirium. Early mobilization can rapidly induce haemodynamic changes, activate the body's responses, and improve patients’ mental state and level of alertness; furthermore, adopting a seated or even standing position at the bedside can enhance patients’ confidence in their recovery and their sense of active participation. Research by Burtin et al. (29) indicates that early mobilization activities for ARDS patients during their ICU stay not only effectively prevent muscle atrophy but also help patients build confidence in overcoming their illness. A study by He Xiaoyuan et al. (32) also confirmed that early mobilization can significantly reduce the incidence of delirium in ARDS patients; these findings are consistent with the results of the present study.

#### 4.2.4 The impact of early mobilization on the incidence of ICU-AW

The study findings indicate that early mobilization in patients with ARDS can reduce the incidence of ICU-AW, consistent with the findings of Zhao Guimei et al. (34). ICU-AW is a common and serious complication in patients with ARDS, and its pathogenesis is closely associated with the inflammatory response and increased muscle catabolism. Early mobilization can modulate the pro-inflammatory–anti-inflammatory balance mediated by interleukin-6 (IL-6), activate muscle cell regeneration signalling pathways, enhance the anabolic rate of skeletal muscle protein synthesis, and improve microcirculatory perfusion and tissue oxygen utilisation (28), thereby maintaining motor function and preventing disuse muscle atrophy. Research by Jiang Fangzheng et al. (6) also indicates that daily arousal combined with early functional mobilization can significantly reduce the risk of ICU-AW in mechanically ventilated patients.

#### 4.2.5 The effect of early mobilization on functional independence scores (Barthel Index)

Research has found that early mobilization in patients with ARDS can improve their functional independence scores (Barthel Index). Due to prolonged bed rest, mechanical ventilation and the effects of ICU-AW, patients with ARDS often experience a marked decline in their ability to perform activities of daily living; some patients are even unable to perform basic activities of daily living independently, which severely affects their quality of life and social reintegration following discharge. Through regular muscle contraction exercises, patients can enhance skeletal muscle strength and endurance, thereby supporting basic functional abilities such as sitting, standing and walking (18); through position changes and balance training, they can improve postural control and coordination, thereby reducing the risk of falls (35); and through daily training, they can promote neuromuscular coordination and the reconstruction of movement patterns, thereby accelerating the recovery of activities of daily living (36). A randomised controlled trial conducted by Schaller et al. (37) demonstrated that early goal-oriented activity intervention can significantly improve Barthel Index scores in patients with ARDS and enhance their functional status at discharge. A systematic review by Tipping et al. (38), which included 13 studies, showed that early mobilization intervention can effectively improve the level of functional independence in ARDS survivors; the findings of these studies are consistent with those of the present study.

#### 4.2.6 The impact of early mobilization on the incidence of complications

Research findings indicate that early mobilization in patients with ARDS can reduce the incidence of complications. Due to the critical nature of their condition, patients with ARDS often require prolonged mechanical ventilation, sedation and bed rest; whilst these treatments are life-saving, they also significantly increase the risk of various complications. Common clinical complications include ventilator-associated pneumonia, deep vein thrombosis, pressure ulcers, muscle atrophy and joint contractures. These complications not only prolong hospital stays and increase healthcare costs, but also seriously affect patients’ prognosis and long-term quality of life (39). Early mobilization increases tidal volume and lung compliance, promotes the clearance of airway secretions and improves the ventilation/ blood flow ratio, thereby effectively preventing ventilator-associated pneumonia and atelectasis (40); regular muscle contractions promote venous return, reduce blood stasis and lower the risk of deep vein thrombosis (41); and position changes and active movement alleviate sustained pressure on local tissues, improve microcirculatory perfusion, and prevent pressure ulcers and joint contractures (42). A systematic review by Zhao et al. (2), which included 12 RCTs, showed that early mobilization intervention significantly reduced the incidence of ventilator-associated pneumonia and deep vein thrombosis in patients with ARDS, consistent with the findings of this study.

### 4.3 Clinical heterogeneity

Although all included studies described their intervention as “early mobilization,” there was substantial heterogeneity in the type of the mobilization protocols across the seven RCTs. This clinical heterogeneity should be carefully considered when interpreting the pooled effect estimates, as different mobilization modalities may exert differential effects on patient outcomes.

The specific early mobilization varied considerably among the included studies. Two studies (19, 20) employed **phased pulmonary rehabilitation**, which primarily focused on breathing exercises, airway clearance, and gradual position changes. In contrast, three studies (21, 23, 24) implemented **conventional physical and occupational therapy**, including passive range-of-motion exercises, active-assisted training, and progressive resistance exercises. One study (22) utilised **NMES** as the primary mobilisation modality, and one (18) adopted a **goal-directed, progressive mobilisation protocol** that included sitting on the edge of the bed, standing, and walking. These different modalities likely target distinct physiological pathways: pulmonary rehabilitation may primarily improve respiratory mechanics and secretion clearance, while active physical therapy may more effectively preserve muscle mass and prevent ICU-AW. The single study that used NMES may have had limited effects on functional outcomes such as the Barthel Index, as passive electrical stimulation does not engage the neuromuscular system in the same way as active exercise. Consequently, pooling these diverse modalities under a single “early mobilization” label may have introduced substantial clinical heterogeneity and may have attenuated or exaggerated the true pooled effect estimates.

Preclinical evidence has demonstrated that early therapeutic mobilization attenuates neutrophilic lung injury and skeletal muscle wasting through modulation of the systemic neutrophil chemokine response, particularly by reducing plasma Granulocyte Colony-Stimulating Factor(G-CSF) concentrations and limiting alveolar neutrophil infiltration (43). Specifically, pulmonary mobilization primarily improves respiratory mechanics and promotes airway clearance, whereas active physical therapy more effectively preserves muscle mass and prevents ICU-AW by inhibiting MuRF1-mediated limb and respiratory muscle atrophy (44). Notably, passive electrical stimulation and active mobilization activate the neuromuscular system through fundamentally different mechanisms: the former triggers muscle contraction via peripheral electrical stimulation of motor axons, whereas the latter involves intact neuromuscular control loops and central motor integration (45). A RCT demonstrated that the addition of NMES to early mobilization significantly improved Barthel Index scores and quality of life, with effects sustained up to six months post-discharge, suggesting a potential additive effect of the two intervention mobilizations (46).

The severity of ARDS, classified according to the Berlin definition, varied across the included studies, encompassing patients with mild to severe disease (47). Those with more severe ARDS typically required deeper sedation and more intensive respiratory support, which may have limited their capacity to participate in active mobilization and thereby attenuated the observed treatment effects. Ventilation strategies also differed across studies: pressure support ventilation, which preserves spontaneous breathing, may facilitate patient cooperation during active exercises, whereas controlled ventilation combined with deep sedation may restrict patients to passive mobilization only (48). Substantial variation in sedation practices was also noted; some studies employed daily sedation interruption to enhance patient participation in physical therapy, whereas others did not specify sedation protocols (21). Lighter sedation strategies have been associated with greater mobilization engagement and more favourable functional outcomes. Beyond these clinical factors, broader ICU mobilization contexts,such as the availability of dedicated physical therapists, nurse-to-patient ratios, and institutional culture regarding early mobilization, may have differed across study settings, potentially influencing both the feasibility and effectiveness of the mobilization (18). However, these contextual factors were rarely reported in sufficient detail to permit quantitative analysis (49). Given the absence of individual patient-level data, subgroup analyses stratified by these clinical parameters were not feasible (50). Consequently, whether the benefits of early mobilization differ across ARDS severity levels, ventilation modes, or sedation strategies remains unclear and warrants further investigation.

### 4.4 Long-term patient-centered outcomes

Although our meta-analysis demonstrated significant short-term benefits of early mobilization in ARDS patients, long-term patient-centered outcomes—including health-related quality of life, sustained physical function, and neurocognitive recovery after hospital discharge,remain inadequately addressed in the current evidence base (51, 52). ARDS survivors frequently experience post-intensive care syndrome (PICS), a constellation of persistent impairments encompassing physical dysfunction, cognitive impairment, and mental health disorders that can persist for years after discharge and substantially impair quality of life and social reintegration (53, 54). However, the role of early mobilization in mitigating these long-term consequences remains uncertain, as most existing RCTs including those in our review,have focused primarily on short-term physiological and functional outcomes during the ICU stay, with limited follow-up beyond discharge (21).

### 4.5 Limitations and future directions

This study included a limited number of publications and had a small sample size; the absence of prospective protocol registration on the PROSPERO international registry before literature retrieval and data extraction. This deficiency arose from insufficient awareness of PRISMA-P and prospective registration standards in the early design stage of our research project. The GRADE assessment showed that the certainty of evidence for most outcomes was low to moderate, primarily due to the limited number of studies, risk of bias, and imprecision. Therefore, the pooled estimates should be interpreted with caution, and further high-quality RCTs are warranted to confirm these findings. Only seven eligible RCTs were included, which does not meet the minimum 10-study threshold required for valid funnel plots, Egger's test and Begg's test. Unpublished small trials with neutral or non-significant results of early mobilization may not be captured by our retrieval strategy, which may lead to overestimation of the pooled beneficial effects of early mobilization for ARDS patients.Only Chinese and English-language publications were included, which may have resulted in incomplete coverage, and grey literature was not included; It is unclear whether the few included studies employed blinding or concealed allocation, resulting in a limited number of high-quality studies being included, which may, to some extent, affect the credibility of the research. It is recommended that clinical researchers prioritise the implementation of blinding and endeavour to adhere to the CONSORT guidelines for reporting clinical RCTs to improve the quality of reporting; Although early mobilization was employed in all intervention groups, the types of activities used varied across studies, including passive mobilization, active training, neuromuscular electrical stimulation and phased pulmonary rehabilitation. The timing of initiation, duration and frequency of early mobilization also differed between studies. There was substantial heterogeneity in the early mobilization protocols across all included studies, which further limits the clinical applicability of the findings of the present analysis. Accordingly, the pooled estimates from this study should be interpreted as an overview of the average effects of diverse mobilization strategies, rather than precise effect estimates for any single precisely defined intervention. Therefore, the results of this study cannot be used to recommend that one specific type of mobilization is superior to others.Furthermore, as the included studies all involved patients with ARDS without further stratification by disease severity, it was not possible to conduct stratified analyses for patients at different severity levels, which may affect the reliability of the analysis results to some extent; Although early mobilization was implemented in all intervention groups, the specific exercise modalities employed varied across studies, as did the timing and frequency of patients’ early mobilization; furthermore, this study did not conduct follow-up on long-term outcome measures following patient activity and paid little attention to patients’ long-term quality of life, which may affect the reliability of the analysis results to some extent; As long-term outcome measures were not included, it is not possible to comprehensively assess the impact of early mobilization on patients’ long-term rehabilitation.

It is therefore recommended that future multicentre, large-sample RCTs be conducted to demonstrate the value and efficacy of early mobilization in patients with ARDS; Strictly standardise early mobilization protocols and procedures, clearly defining the timing, type, duration, frequency and intensity of the programme; implement early mobilization for patients with ARDS at different levels of severity and analyse the differentiated clinical outcomes; provide a detailed description of the trial methodology, intervention steps, quality control and measurement of outcome measures for both the control and intervention groups to obtain authentic and reliable trial data; and, at the same time, extend the follow-up period, refine the outcome measure system and comprehensively assess the long-term benefits of early mobilization.

## 5 Conclusion

This study found that early mobilization in patients with ARDS can effectively reduce the incidence of ICU-AW and delirium, shorten the duration of mechanical ventilation, ICU stay and total hospitalisation, improve functional independence scores, and reduce the incidence of complications; it is therefore recommended that this practice be promoted in clinical settings. However, due to limitations in the number of included studies, sample size and methodological quality, the conclusions of this study require further validation through multicentre, large-sample, methodologically rigorous randomised controlled trials to determine the efficacy of early mobilization in patients with ARDS and to identify the optimal implementation strategy.

## Data Availability

The original contributions presented in the study are included in the article/Supplementary Material, further inquiries can be directed to the corresponding author.
