# Supplementary material for: Efficacy and safety of early mobilization in patients with ARDS: a systematic review and meta-analysis
Source: Front Med (Lausanne). 2026 Sep 21;13:1928774. doi: 10.3389/fmed.2026.1928774 (PMC13635334; doi:10.3389/fmed.2026.1928774)
Supplement: Supplementary file 1 [file Datasheet1.docx]

Supplementary Material

# 1 Supplementary Figures and Tables

TABLE 1 PubMed search strategy.

| Steps | Search strategies |
| --- | --- |
| #1 | "Respiratory Distress Syndrome"[Mesh] OR  ARDS[Title/Abstract] OR acute respiratory distress  syndrome[Title/Abstract] OR adult acute respiratory distress  syndrome[Title/Abstract] OR lung injury[Title/Abstract] OR  acute hypoxemic respiratory failure[Title/Abstract] |
| #2 | "Early Ambulation"[Mesh] OR early  ambulation[Title/Abstract] OR early  mobilization[Title/Abstract] OR early activity[Title/Abstract]  OR early physical therapy[Title/Abstract] OR early  physiotherapy[Title/Abstract] OR early  rehabilitation[Title/Abstract] OR early exercise[Title/Abstract]  OR early training[Title/Abstract] OR accelerated  ambulation[Title/Abstract] |
| #3 | #1 AND #2 |

FIGURE1

Flow diagram of the study selection process.

TABLE 2 Basic characteristics of the included studies.

| Study | Country | Sample size | Age | Interventions | Outcome indicators |
| --- | --- | --- | --- | --- | --- |
| Hodgson et al., | Australia and New Zealand | C:21  E:29 | C: 53±15  E: 64±12 | C: Routine care + physiotherapy (primarily passive movement)  E:Early Goal-Directed Mobilisation (EGDM):Active functional rehabilitation based on the ICU Activity Scale (IMS), carried out at the highest tolerable level for one hour daily, implemented in stages | ②⑥⑧⑨ |
| Moss et al., | United States | C:61  E:59 | C: 49±15  E: 56±14 | C:Routine physiotherapy: three sessions per week during hospitalisation (primarily passive exercises); exercise guidance is provided following discharge  E: Intensive physiotherapy: Once daily during hospitalisation (30 minutes per session in the ICU; 60 minutes per session in general wards or as an outpatient), followed by three sessions per week at home or as an outpatient after discharge, for a total of 28 days, including breathing exercises, joint mobilisation, muscle strengthening and functional rehabilitation | ②⑥ |
| Schweicker et al., | United States | C:55  E:49 | C: 54.4（46.5-66.4）  E: 57.7（36.3-69.1） | C: Discontinuation of sedation + physiotherapy/occupational therapy as required (no standardised protocol)  E: Interruption of sedation + early physiotherapy/occupational therapy: Following the daily interruption of sedation, progress gradually from passive joint mobilisation to sitting at the bedside, transfers and walking, continuing until discharge or the restoration of independent function | ①②④⑤⑥ |
| Patel et al., | United States | C:39  E:44 | C: 58.4（50.8-71.5）  E: 58.2（49.7-66.6） | C: Non-invasive ventilation (NiV) via face mask + routine mobilisation: rehabilitation (primarily passive exercises) was carried out on 43.9% of the days during NiV  E: Non-invasive ventilation (NiV) via a helmet + early mobilisation: rehabilitation (limb exercises, sitting up in bed, standing and walking) was carried out on 63.6% of the days during NiV. | ②③④⑤⑥⑦ |
| Fan et al., | China | C:30  E:29 | C:47.53±7.56  E:47.52±8.62 | C: General Rehabilitation：Passive/active limb exercises, bedside sitting exercises and breathing exercises, 1 hour per day  E:Conventional rehabilitation + neuromuscular electrical stimulation (NMES)：To be commenced 48 hours after admission to the ICU, for 1 hour per day, for 7 consecutive days, to stimulate the rectus femoris and tibialis anterior muscles | ①②④⑤⑥ |
| Liang et al., | China | C:40  E:38 | C:41.43±11.72  E:42.18±11.62 | C: Standard treatment：Mechanical ventilation + medication + basic nursing care  E:Early-stage,phased pulmonary rehabilitation：3 stages, including low-tidal-volume ventilation, prone positioning, limb movement and breathing exercises, 4–6 times a day | ①②③⑦ |
| Wu et al., | China | C:39  E:39 | C:51.28 ±5. 34  E:50.72 ± 5. 51 | C: Conventional ventilation (non-ASV mode) + basic rehabilitation (passive movement, simple breathing exercises)  E:Early-stage, phased pulmonary rehabilitation + Adaptive Support Ventilation (ASV): Rehabilitation comprises breathing exercises, muscle strengthening and functional rehabilitation, twice daily; the ventilation mode is ASV | ②⑦ |

C, Control group; E, Experimental group;①Duration of mechanical ventilation;②Length of stay in the ICU;③Incidence of delirium;④Incidence of ICU-acquired delirium;⑤Functional independence score (Barthel Index);⑥Total length of hospital stay;⑦Incidence of complications.

TABLE 3 GRADE Assessment of meta-analytic results.

| Outcome | Number of studies(Participants) | Effect estimate | Heterogeneity | GRADE Certainty | Reasons for Downgrading |
| --- | --- | --- | --- | --- | --- |
| Duration of mechanical ventilation | 7 (572) | MD −2.40（−2.96，−1.84） | 70% (excluded: 19%) | ⊕⊕⊕◯ MODERATE | Risk of bias (−1); Publication bias (−1) |
| Length of stay in the ICU | 5(416) | MD −3.36（−4.21，−2.52 | 34% | ⊕⊕⊕◯ MODERATE | Risk of bias (−1); Publication bias (−1) |
| Incidence of delirium | 3(265) | OR 0.51（0.28，0.91） | 0% | ⊕⊕◯◯ LOW | Risk of bias (−1); Imprecision (−1); Publication bias (−1) |
| Incidence of ICU-AW | 3(246) | OR 0.35（0.19，0.64） | 0% | ⊕⊕⊕◯ MODERATE | Risk of bias (−1); Publication bias (−1) |
| Functional Independence Score (Barthel Index) | 3(246) | MD 17.27（11.18，23.35） | 85% (excluded: 0%) | ⊕⊕⊕◯ MODERATE | Risk of bias (−1); Publication bias (−1) |
| Total length of stay | 6(494) | MD −2.74（−3.49，−1.98 | 25% | ⊕⊕⊕◯ MODERATE | Risk of bias (−1); Publication bias (−1) |
| Incidence of complications | 3(239) | OR 0.39（0.17，0.86） | 66% (excluded: 0%) | ⊕⊕◯◯ LOW | Risk of bias (−1); Imprecision (−1); Publication bias (−1); Upgraded for large effect (+1) |

TABLE 4 Methodological quality assessment of included studies.

| Study | Generation of random sequences | Hide allocation | Blinding of participants and investigators | Blinded assessor method | Complete end-game data | Selective reporting of results | Bias from other sources |
| --- | --- | --- | --- | --- | --- | --- | --- |
| Hodgson et al., | Low risk | Not sure | Not sure | Not sure | Low risk | Low risk | Low risk |
| Moss et al., | Not sure | Not sure | Not sure | Not sure | Low risk | Low risk | Low risk |
| Schweicker et al., | Low risk | Low risk | Not sur | Low risk | Low risk | Low risk | Low risk |
| Patel et al., | Low risk | Not sure | Not sure | Not sure | Low risk | Low risk | Low risk |
| Fan et al., | Low risk | Low risk | Not sure | Not sure | Low risk | Low risk | Low risk |
| Liang et al., | Not sure | Not sure | Not sure | Not sure | Low risk | Low risk | Low risk |
| Wu et al., | Not sure | Not sure | Not sure | Not sure | Low risk | Low risk | Low risk |

FIGURE 2-3 Risk-of-bias assessment of the included studies.

FIGURE 4 A forest plot showing the effect of early activity on the duration of mechanical ventilation.

FIGURE 5 A forest plot showing subgroup analysis by mobilization type

FIGURE 6 A forest plot showing the effect of early activity on the length of stay in the ICU.

FIGURE 7 A forest plot showing the effect of early activity on the incidence of delirium.

FIGURE 8 A forest plot showing the effect of early activity on the incidence of ICU-AW.

FIGURE 9 A forest plot showing the effect of early activity on the functional Independence Score (Barthel Index).

FIGURE 10 A forest plot showing the effect of early activity on the total length of stay.

FIGURE 11 A forest plot showing the effect of early activity on the incidence of complications.
